# Effects of sodium citrate and acid citrate dextrose solutions on cell counts and growth factor release from equine pure-platelet rich plasma and pure-platelet rich gel

**DOI:** 10.1186/s12917-015-0370-4

**Published:** 2015-03-14

**Authors:** Carlos E Giraldo, María E Álvarez, Jorge U Carmona

**Affiliations:** Grupo de Investigación Terapia Regenerativa, Departamento de Salud Animal, Universidad de Caldas, Manizales, Colombia

**Keywords:** Anticoagulant, Horse, Platelet concentrate, Transforming growth factor beta 1, Platelet derived growth factor isoform BB, Regenerative therapy

## Abstract

**Background:**

There is a lack information on the effects of the most commonly used anticoagulants for equine platelet rich plasmas (PRPs) elaboration on cell counts and growth factor release from platelet rich gels (PRGs). The aims of this study were 1) to compare the effects of the anticoagulants sodium citrate (SC), acid citrate dextrose solution A (ACD-A) and ACD-B on platelet (PLT), leukocyte (WBC) and on some parameters associated to platelet activation including mean platelet volume (MPV) and platelet distribution width (PDW) between whole blood, pure PRP (P-PRP) and platelet-poor plasma (PPP); 2) to compare transforming growth factor beta 1 (TGF-β_1_) and platelet-derived growth factor isoform BB (PDGF-BB) concentrations in supernatants from pure PRG (P-PRG), platelet-poor gel (PPG), P-PRP lysate (positive control) and plasma (negative control); 3) to establish the possible correlations between all the studied cellular and molecular parameters.

**Results:**

In all cases the three anticoagulants produced P-PRPs with significantly higher PLT counts compared with whole blood and PPP. The concentrations of WBCs were similar between P-PRP and whole blood, but significantly lower in PPP. The type of anticoagulant did not significantly affect the cell counts for each blood component. The anticoagulants also did not affect the MPV and PDW parameters. Independently of the anticoagulant used, all blood components presented significantly different concentrations of PDGF-BB and TGF-β_1_. The highest growth factor (GF) concentrations were observed from P-PRP lysates, followed by PRG supernatants, PPP lysates, PPG supernatants and plasma. Significant correlations were observed between PLT and WBC counts (ρ = 0.80), PLT count and TGF-β_1_ concentration (ρ = 0.85), PLT count and PDGF-BB concentration (ρ = 0.80) and PDGF-BB and TGF-β_1_ concentrations (ρ = 0.75). The type of anticoagulant was not correlated with any of the variables evaluated.

**Conclusions:**

The anticoagulants did not significantly influence cell counts or GF concentrations in equine PRP. However, ACD-B was apparently the worst anticoagulant evaluated. It is necessary to perform additional research to determine the effect of anticoagulants on the kinetics of GF elution from P-PRG.

## Background

There is an increased use of platelet-rich plasma (PRP) as a treatment for musculoskeletal diseases and severe wounds in horses [1-4]. It was recognized that among the regenerative therapies, platelet-rich plasma (PRP) is an autologous platelet concentrate suspended in plasma that, administrated in the wound site, releases growth factors and promotes the wound healing cascade [2,5]. Platelets contain a pool of growth factors, including transforming growth factor-b (TGF-β), platelet derived growth factor (PDGF) and vascular endothelial growth factor (VEGF), mainly contained in platelet alpha granules [6] that are released after platelet degranulation in the damage site and enhance tissue regeneration by stimulating cell proliferation, increasing extracellular matrix synthesis, promoting vascular ingrowth and reducing catabolic matrix-degrading cytokines such as interleukins and matrix metalloproteinases [5,7].

PRP intended for regenerative proposes may be classified as: pure-platelet rich plasma (P-PRP) or leukoreduced PRP, leukocyte- and platelet-rich plasma (L-PRP) and platelet rich fibrin (PRF). P-PRP and L-PRP are obtained in a liquid form by using anticoagulants [8]. PRF is a second generation platelet concentrate, which does not require anticoagulant for its elaboration. In horses, P-PRP displays slightly higher platelet counts (1.3 - 4.0 fold) and leukocyte (WBC) counts (0.5 - 2.0 fold) than whole blood, whereas L-PRP has increased platelet (5 fold) and leukocyte (3 - fold or more) counts when compared with whole blood. There is not a complete consensus regarding the role of leukocyte concentrations in PRP [2]. However, *in vitro* evidence suggests that leukoreduced PRP could be more suitable for the treatment of tendon and soft tissue injuries in horses, as this substance induces tendon anabolism and decreases the expression of catabolic cytokines when compared with L-PRP [9].

Although PRP (either L-PRP or P-PRP) is employed as a promising treatment in equine practice [5], there are some controversial issues that should solved to improve the clinical use of this substance in horses and other animals. There are a plethora of PRP products and PRP-associated technologies that are used in human and equine practices [2,8,10]. However, little is known regarding the cellular and molecular quality of these substances, as they are influenced by intrinsic factors that are dependent on the patient, such as gender, age, breed [6] and pathological conditions [11], amongst others and by extrinsic factors, such as the type of anticoagulant used [12], the relative centrifugation forces (rcf or g) used for cell concentration [2,13,14], the type and form of the kit used for PRP preparation and the activating substance used for PRP activation and growth factor release [2,15].

Recent equine PRP studies have showed that the cell and growth factor release profiles are influenced by the intrinsic factors of the patients [6]. Furthermore, it has also been observed that activating substances, including calcium salts and thrombin, affect the growth factor release profile from equine PRP [15]. However, there is a lack of information of the effect on the most commonly used anticoagulants for PRP elaboration on cell counts and growth factor release. Although, a human study indicated that acid citrate dextrose solution A (ACD-A) was better than sodium citrate (SC) for PRP preparation [12], there is no information regarding the effect of the type of anticoagulant used for PRP preparation in horses on cell counts from PRP or growth factor release from PRG.

The aims of this study were: 1) to compare the effects of the anticoagulants SC, ACD-A and ACD-B on platelet and leukocyte counts and platelet activation associated parameters, such as mean platelet volume (MPV) and platelet distribution width (PDW) between whole blood, P-PRP and platelet-poor plasma (PPP); 2) to compare the PDGF-BB and TGF-β_1_ concentrations in the supernatants from pure platelet-rich gel (P-PRG), platelet-poor gel (PPG), P-PRP lysate (positive control) and plasma (negative control); 3) to establish the possible correlations between all the studied cellular and molecular parameters.

The hypothesis of this study was that anticoagulants do not influence cell counts and PDGF-BB and TGF-β_1_ release from equine P-PRP/P-PRG.

## Methods

This study was approved by the Ethical Committee of the Universidad de Caldas.

### Horses

Eighteen clinically normal Argentinean Creole horses (geldings) were used. The horses had a mean age of 12.5 (**±** standard deviation (s.d) 6.3) years old. All the horses were from the same farm, and the owner did know the nature of the study and authorized the blood extraction accordingly.

### Blood collection and preparation of platelet concentrates

From each animal blood samples were collected in triplicate by jugular venipuncture and deposited randomly in tubes with either sodium citrate (SC) (12.35 mg sodium citrate and 2.21 mg citric acid [BD Vacutainer®, Becton Drive, Franklin Lakes, NJ, USA]) or acid citrate dextrose (ACD) solution A (ACD-A) (22.0 g/L trisodium citrate, 8.0 g/L citric acid and 24.5 g/L dextrose [BD Vacutainer®, Becton Drive, Franklin Lakes, NJ, USA]) or ACD solution B (ACD-B) (13.2 g/L trisodium citrate 4.8 g/L citric acid and 14.7 g/L dextrose [BD Vacutainer®, Becton Drive, Franklin Lakes, NJ, USA]).

**Figure 1:**
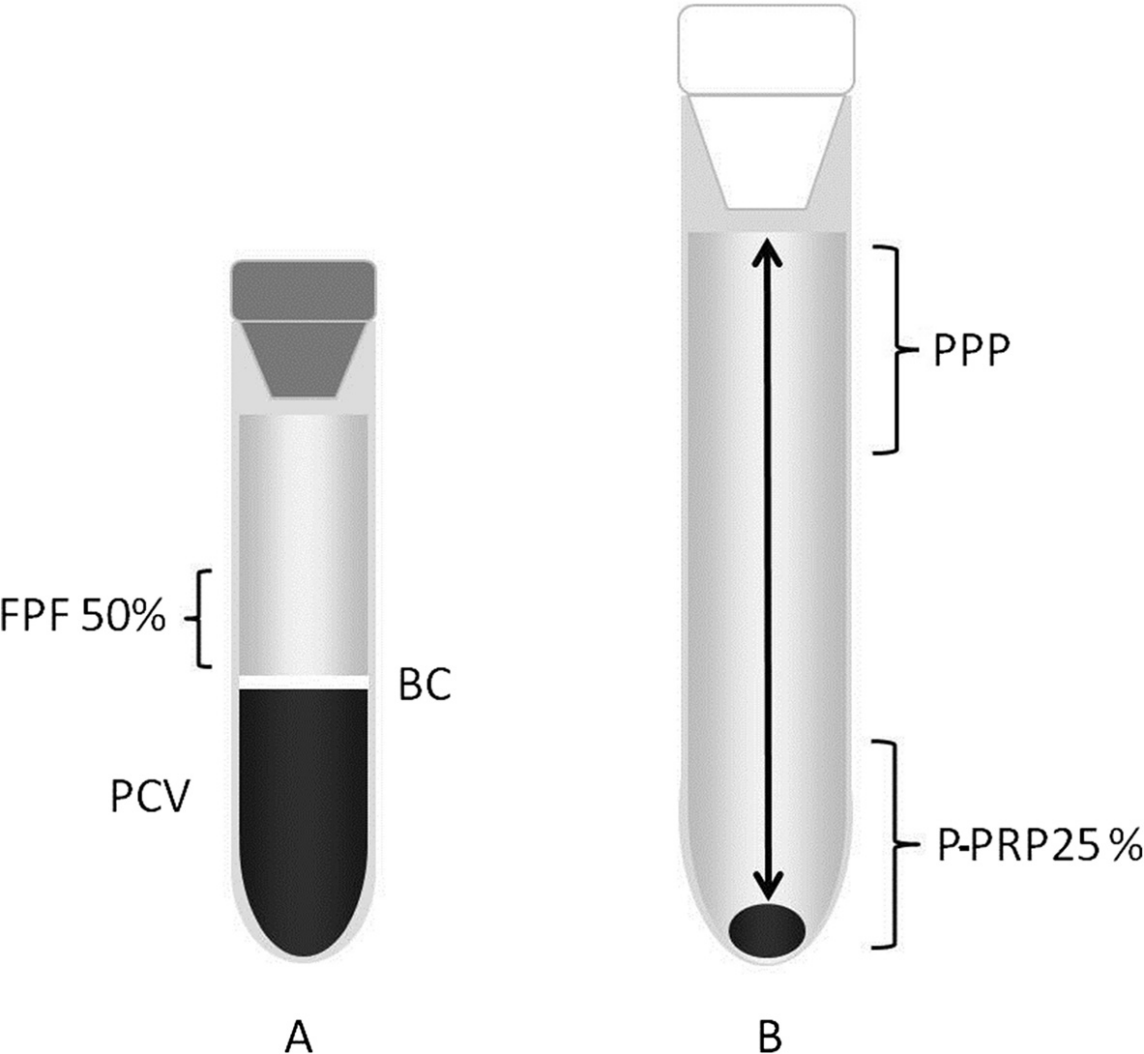
**Schematic representation of the plasma fractions obtained with the tube method protocol.** Left tube **(A)** containing the first fraction of plasma (50%) (PFP) obtained by the single centrifugation tube method. Right tube **(B)** containing platelet-rich plasma (PRP) obtained by the double centrifugation tube method. BC: buffy coat. PCV: packed cell volume.

Tubes with each anticoagulant were randomly processed for P-PRP production. The total whole blood used for P-PRP preparation using each anticoagulant varied between 110 and 140 mL. Briefly, after centrifugation at 120 *g* for 5 min, the first 50% of the top supernatant plasma fraction, adjacent to the buffy coat, was collected. This fraction was then centrifuged at 240 *g* for 5 min and the bottom quarter fraction was collected [16]. This fraction was considered to be P-PRP. The upper plasma fraction P-PRP was considered to be PPP (Figure 1). Plasma was obtained by centrifugation from each anticoagulated blood at 3500 *g* for 8 min. The time between blood collection and processing was approximately 1 h. All the samples were deposited and transported from the farm to the laboratory in an icebox.

### Haematological analysis

Complete, automated haemograms (Celltac-α MEK 6450, Nihon Kodhen, Japan) were performed in duplicate for whole blood, P-PRPs and PPPs obtained from each anticoagulant. Platelet (PLT) counts, mean platelet volume (MPV fL), platelet distribution width (PDW %) and total leukocyte (WBC) counts were determined.

### Activation of platelet concentrates

Four hundred μL of a 10% calcium gluconate (CG) solution (9.3 mg/mL) (Ropsohn Therapeutics Ltda®, Bogotá, Colombia) was added to 4 mL of P-PRP or PPP obtained with each anticoagulant to produce the P-PRGs and PPGs, respectively. P-PRGs and PPGs were incubated at 37°C for 3 h to stimulate GF release. Clots were mechanically released from the walls of the tubes and centrifuged at 3500 *g* for 8 min. The resulting supernatant was aliquoted, and frozen at −82°C for later determination of TGF-β_1_ and PDGF-BB concentrations.

### Lysis of platelet concentrates

Samples of 4 mL of P-PRPs and PPPs obtained using each anticoagulant were incubated at 37°C for 15 min with 400 μL of a solution containing 0.5% of a non-ionic detergent (NID) (Triton® X100, Panreac Química, Barcelona, Spain). Platelet concentrates treated with NID were used as a positive control for GF release [11]. Lysates were processed in a similar fashion to supernatants from P-PRGs and PPGs.

### Total protein determination

Total protein (TP) concentration from all the samples were determined using the biuret method (Proteína total (Biuret), BioSystems, Barcelona, Spain) [17], followed by spectrophotometric quantifications.

### Determination of TGF-β_1_ and PDGF-BB concentrations by ELISA

The TGF-β_1_ and PDGF-BB concentrations from the supernatants and lysates of each blood component were determined in duplicate by a sandwich ELISA using commercially available antibodies against human TGF-β_1_ (Human TGF-β1, DY240E, R&D Systems, Inc., Minneapolis, MN USA) and PDGF-BB (Human PDGF-BB, DY220, R&D Systems, Inc.). Both ELISAs were performed according to the manufacturer’s instructions. Readings were performed at 450 nm. Both ELISAs were determined with human antibodies because there is a high homology of these growth factors between equines and humans [18,19]. Further, several equine PRP studies have validated these ELISA kits [6,14-16].

### Statistical analysis

Data were analysed using commercial software (SPSS 18.0, IBM, Chicago, IL, USA). Data were initially assessed for normality (goodness of fit) by a Shapiro-Wilk test and a direct plot analysis of each evaluated variable. When the variables had a normal distribution (Shapiro-Wilk test, P > 0.05), they were presented as means (**±** s.d.) and evaluated by parametric tests (e.g., Student’s *t*-test for paired samples, and one way analysis of variance (ANOVA) and Tukey’s test (for *post-hoc* paired comparisons). Non-parametric variables (Shapiro-Wilk test, P <0.05) were presented as medians (interquartile range -IR-) and evaluated using a Kruskal-Wallis test followed, when necessary, by a Mann–Whitney U-test. A Wilcoxon test was used for non-related paired comparisons. All the variables were analysed for general and specific correlations using a Spearman (*r*_s_) test. A P value ≤0.05 was considered to be significant for all tests.

## Results

### Haematological findings

In all cases, the three anticoagulants produced P-PRPs with significantly (P < 0.001) higher PLT counts compared with whole blood and PPP. The concentrations of WBCs were similar between P-PRP and whole blood, but significantly (P < 0.001) lower in PPP. The type of anticoagulant did not significantly affect the cell counts for each blood component. The anticoagulants also did not affect the MPV and PDW parameters. However, in general, these platelet activation parameters were significantly higher in P-PRP than in PPP. A summary of the haematological results is shown in Table 1.

**Table 1:** **Means (± s.d) of the haematological variables for each blood component obtained with every anticoagulant**

**Variable**	**Anticoagulant**								
	**Sodium citrate (SC)**			**ACD-A**			**ACD-B**		
	**Whole blood**	**P-PRP**	**PPP**	**Whole blood**	**P-PRP**	**PPP**	**Whole blood**	**P-PRP**	**PPP**
PLT (10^3^/μL)	143.8 (19.4)^a,b^	390.6 (57.6)^c^	111.0 (22.6)	137.0 (21.3)^a^	399.1 (62.8)^c^	112.6 (23.7)	137.1 (25.4)^a^	398.5 (48.0)^c^	111.2 (18.5)
MPV (fL)	3.8 (0.4)	4.1 (0.6)^b^	3.6 (0.3)	3.8 (0.4)	4.2 (0.6)^b^	3.7 (0.4)	3.8 (0.4)^d^	4.2 (0.5)^b^	3.7 (0.4)
PDW (%)	16.5 (0.5)^c^	16.8 (0.5)^c^	17.8 (0.5)	16.3 (0.5)^c^	16.5 (0.6)^c^	17.6 (0.7)	16.1 (0.6)^c^	16.7 (0.6)^c^	17.8 (0.7)
WBC (10^3^/μL)*	8.4 (1.7)^c,d^	9.5 (3.0)^c^	0.1 (0.0)	7.9 (2.0)^c,d^	9.8 (5.0)^c^	0.1 (0.0)	8.3 (2.3)^e^	10.6 (4.0)^c^	0.1 (0.0)

ACD: acid citrate dextrose (solution-A,-B); P-PRP: pure platelet-rich plasma; PPP: platelet-poor plasma; PLT, platelets; MPV: mean platelet volume; PDW: platelet distribution width; WBC: white blood cells. Lower-case letters represent significant differences between blood components for every independent anticoagulant. Blood components significantly different with a: P-PRP (P <0.001); b: PPP (P <0.05); c: PPP (P <0.001); d: P-PRP (P <0.05); and e: P-PRP PPP (P <0.001); *Data are presented as medians (interquartile range (IR).

### Growth factor release from blood components

**Figure 2:**
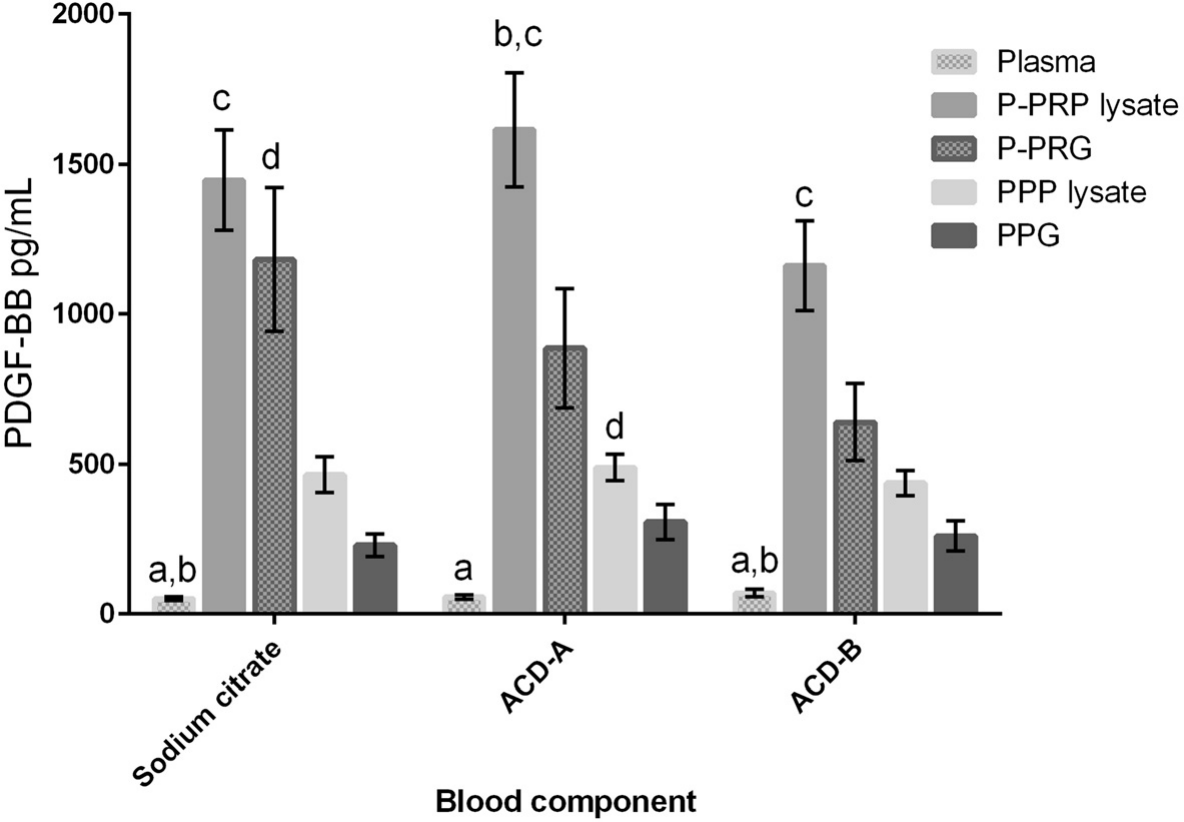
**Means (standard error of the mean (s.e.m)) of PDGF-BB concentration (pg/mL) in the different blood components.** Lower-case letters denote significant differences between blood components for every independent anticoagulant. Sodium citrate (SC): blood component significantly different with a: pure platelet-rich plasma (P-PRP) lysate and platelet poor plasma (PPP) lysate (P <0.001); b: pure platelet-rich gel (P-PRG) and platelet poor gel (PPG) (P <0.05); c: PPG and PPP lysate (P <0.001); and d: PPG (P <0.05). Acid citrate dextrose solution A (ACD-A): blood component significantly different with a: P-PRP lysate, PPP lysate, P-PRG and PPG (P <0.001); b: PPG and PPP lysates (P <0.001); and c: P-PRG (P <0.05). ACD-B: blood component significantly different with a: P-PRP and PPP lysates (P <0.001); b: P-PRG and PPG (P <0.05); and c: PPG and PPP lysates (P <0.001).

**Figure 3:**
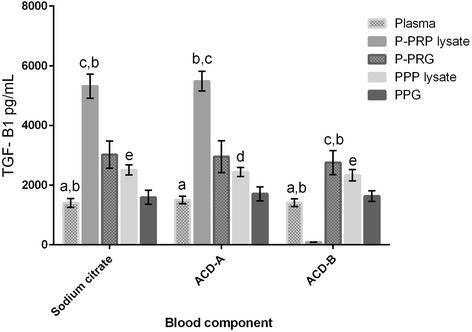
**Means (s.e.m) of TGF-β**_1_ **concentration (pg/mL) in the different blood components.** Lower-case letters denote significant differences between blood components for every independent anticoagulant. SC: blood component significantly different with a: P-PRP lysate (P <0.001); b: P-PRG and PPP lysates (P <0.05); c: PPG and PPP lysates (P <0.001); d: P-PRG (P <0.05); and e: PPG (P <0.05). ACD-A: blood component significantly different with a: P-PRP and PPP lysates (P <0.001); b: PPG and PPP lysates (P <0.001); c: P-PRG (P <0.05); and d: PPG (P <0.05). ACD-B: blood component significantly different with a: PRP and PPP lysates (P <0.001); b: P-PRG (P <0.05); c: PPG and PPP lysates (P <0.001); d: P-PRG (P <0.001); and e: PPG (P <0.05).

Independently of the anticoagulant used, all blood components presented significantly different concentrations of PDGF-BB and TGF-β_1_. The highest GF concentrations were observed from P-PRP lysates, followed by PRG supernatants, PPP lysates, PPG supernatants and plasma (Table 2). However, when data were plotted, a statistical trend (P = 0.20) was observed for PDGF-BB concentrations in P-PRG from SC in comparison with ACD-B (Figure 2). In contrast, this trend was not observed for TGF-β_1_ released from P-PRG (Figure 3).

**Table 2:** **Means (± s.d) of the TGF-β**_1_ **and PDGF-BB concentrations (pg/mg of total protein (TP)) in every blood component obtained with every anticoagulant**

**Variable**	**Blood component**				
	**Plasma**	**P-PRP lysate**	**P-PRG**	**PPP lysate**	**PPG**
**SC**					
PDGF-BB (pg/mg of TP)	0.9 (0.6)^a,b^	25.2 (14.4)^c^	19.0 (29.4)^d^	8.3 (5.4)^d^	4.5 (5.5)
TGF-β1(pg/mg of TP)*	26.8 (10.4)^a,e^	90.7 (30.7)^c,e^	54.5 (33.1)	45.2 (10.3)^d^	29.2 (17.1)
**ACD-A**					
PDGF-BB (pg/mg of PT)	1.0 (0.6)^a,b^	28.2 (20.1)^c^	11.3 (30.6)	8.2 (5.0)	5.8 (8.0)
TGF-β1(pg/mg of TP)*	30.0 (8.7)^a^	101.5 (31.2)^c,e^	56.4 (39.1)	47.8 (12.2)	33.3 (17.9)
**ACD-B**					
PDGF-BB (pg/mg of TP)	1.1 (0.9)^a^	18.4 (13.4)^b^	6.6 (17.3)	7.2 (4.1)	4.8 (6.5)
TGF-β1(pg/mg of TP)*	27.5 (10.0)^c^	87.8 (23.0)^d^	50.8 (31.2)	44.6 (12.9)	31.3 (12.7)

*Data are presented as medians (IR). P-PRG: pure platelet-rich gel; PPG: platelet-poor gel. Lowercase letters represent independent significant differences for every blood component obtained with a specific anticoagulant. SC: blood component different with a: P-PRP and PPP lysates (P <0.001); b: P-PRG and PPG (P <0.05); c: PPP lysate and PPG (P <0.001); d: PPG (P <0.05); and e: P-PRG (P <0.05). ACD-A: blood component different with a: P-PRP and PPP lysates (P <0.001); b: P-PRG and PPG (P <0.05); c: PPP lysate and PPG (P <0.001); d: PPG; and e: P-PRG (P <0.05). ACD-B: blood component different a: all blood components (P <0.05); b: PPP lysate and PPP (P <0.05); c: P-PRP lysate (P <0.05); d: PPP lysate and PPG (P <0.05).

### Correlations

Significant correlations were observed between PLT and WBC counts (ρ = 0.80, P <0.01), PLT counts and TGF-β_1_ concentrations (ρ = 0.85, P <0.01), PLT counts and PDGF-BB concentrations (ρ = 0.80, P <0.01) and PDGF-BB and TGF-β_1_ concentrations (ρ = 0.75, P <0.01). The type of anticoagulant was not correlated with any of the variables evaluated.

## Discussion

To our knowledge, this is the first study to evaluate the effects of several anticoagulants used for producing equine PRP as a regenerative therapy. The cellular results from this study were similar to those previously reported by other equine PRP studies, in which the double centrifugation tube method was used [6,13].

The parameters associated with platelet activation, such as MPV and PDW were not affected by the type of anticoagulant evaluated in this study. However, MPV and PDW values were significantly lower in PPP when compared with whole blood and P-PRP, although they remained within the normal physiological values for this species [6]. It is known, that ACD is a very good anticoagulant, compared to SC for preserving the structural and physiological properties of platelets after two or more hours of blood collection [20]. From a regenerative medicine perspective, ACD should be used to conserve PLT integrity in situations in which the processing (and transporting) of the blood samples could take two or more hours before the PRP can be used.

Although the type of anticoagulant did not significantly influence the PDGF-BB and TGF-β_1_ concentrations in the different blood components in the present study, there was a better apparent concentration of PDGF-BB in the blood components processed with SC, followed by ACD-A and ACD-B. In contrast, when TGF-β_1_ concentrations were evaluated, there were better apparent concentrations of this GF in the blood components processed with ACD-A, followed by SC and ACD-B. The same finding was reported for human PRP obtained with ACD-A and SC [12].

Notably, ACD-B had a very negative influence on GF concentrations when compared with the other anticoagulants. It is possible that the type of anticoagulant influenced (albeit not significantly) the release patterns of both GFs from all P-PRGs, as PDGF-BB release was substantially larger from platelet clots processed with SC in comparison with ACD-A and ACD-B. In contrast, TGF-β_1_ release was more uniform (50% of the concentration with respect to P-PRP lysates) from the P-PRGs obtained with any of the three anticoagulants.

Despite the intriguing results observed regarding GF release from P-PRGs, the present study may have had some methodological limitations. For instance, perhaps measuring GF release at a single time point is not appropriate for determining the exact influence of the anticoagulants on GF release from P-PRGs [15]. In this situation, it is imperative to perform a study that evaluates the elution kinetics of both GFs at several time points. This study is necessary to determine whether the type of anticoagulant could produce GF loss (degradation) or GF absorption in the P-PRGs.

Many P-PRPs produced by manual tube protocols in different species (including equines) are performed with commercial vacuum tubes for *in vitro* diagnoses, not for therapeutic purposes [6,13,21,22]. This is a well-manifested concern by researches defending the use of commercial kits for producing platelet concentrates [23]. However, in the experience of the authors, the only problem with using commercial tubes with anticoagulants for equine PRP processing is that the PLT collection efficiency is very low [6,16]. The use of many tubes during PRP preparation could be associated with a risk of bacterial contamination [24] and with a major time expenditure for PRP processing [2]. However, it is well recognized that the main source for bacterial contamination during PRP processing is the skin of the venipuncture site, not the tubes [24]. In view of these limitations, it is possible that the use of ACD-A tubes could be more suitable for manual PRP processing, as the volume capacity of the tubes is almost 44% greater than that of sodium citrate tubes.

The correlations obtained in this study were similar to those obtained in previous equine PRP studies, which evaluated manual protocols [6,16]. In general, there were moderate to strong correlations between cell (PLT and WBC) counts and GF concentrations. These findings are in agreement with several procedures for obtaining PRP in humans [25], dogs [26] and cattle [27]. The role of WBCs in PRP is controversial because there are data supporting the catabolic effect of these cells in equine tendon explants [9]. However, this situation could be more clinically relevant when L-PRP preparations are used [28]. The authors believe that the number of WBCs concentrated in the P-PRPs from this study could be beneficial for treating tissues because these cells are correlated with GF concentrations, especially TGF-β_1_ [6].

## Conclusions

This study presents new information regarding the effect of the anticoagulants: SC, ACD-A and ACD-B, for the elaboration of equine P-PRP. The results obtained in the study confirm the working hypothesis that the anticoagulants evaluated did not significantly influence cell counts or GF concentrations in equine P-PRP. However, ACD-B was apparently the worst anticoagulant evaluated, because it produced the lower cell counts and GF concentrations when compared with the other two anticoagulants. It is necessary to perform additional research to determine the GF elution kinetics from P-PRGs obtained with the anticoagulants evaluated in this study.

## Abbreviations

ACD-A(B): Acid citrate dextrose solution A(B)
BC: Buffy coat
CG: Calcium gluconate
COL1: Collagen type I
EOS: Eosinophils
FPF: First plasma fraction
GF: Growth factors
WBC: Leukocytes (white blood cells)
L-PRP: Leukocyte-platelet rich plasma
LY: Lymphocytes
MPV: Mean platelet volume
NID: Non-ionic detergent
PLT: Platelet
PC: Platelet concentrates
PDGF-BB: Platelet derived growth factor isoform BB
PDW: Platelet distribution width
PG: Platelet gels
PPG: Platelet poor plasma gel
PPP: Platelet poor plasma
PRF: Platelet rich fibrin
P-PRP: Pure platelet-rich plasma
P-PRG: Pure platelet-rich gel
SC: Sodium citrate
TP: Total protein
TGF-β_1_: Transforming growth factor beta 1
